# Combining Molecular, Imaging, and Clinical Data Analysis for Predicting Cancer Prognosis

**DOI:** 10.3390/cancers14133215

**Published:** 2022-06-30

**Authors:** Barbara Lobato-Delgado, Blanca Priego-Torres, Daniel Sanchez-Morillo

**Affiliations:** 1 Unitat de Genòmica de Malalties Complexes, Institut de Recerca de l’Hospital de la Santa Creu i Sant Pau, IIB Sant Pau, 08041 Barcelona, Spain; blobato@santpau.cat; 2 Department of Automation Engineering, Electronics and Computer Architecture and Networks, Universidad de Cádiz, Puerto Real, 11519 Cádiz, Spain; blanca.priego@uca.es; 3 Biomedical Engineering and Telemedicine Research Group, Universidad de Cádiz, Puerto Real, 11519 Cádiz, Spain; 4 Instituto de Investigación e Innovación Biomédica de Cádiz (INiBICA), 11009 Cádiz, Spain

**Keywords:** cancer, survival analysis, prognosis prediction, patient risk stratification, multimodal data, data integration, Artificial Intelligence, machine learning

## Abstract

**Simple Summary:**

The rise of Big Data, the widespread use of Machine Learning, and the cheapening of omics techniques have allowed for the creation of more sophisticated and accurate models in biomedical research. This article presents the state-of-the-art predictive models of cancer prognosis that use multimodal data, considering clinical, molecular (omics and non-omics), and image data. The subject of study, the data modalities used, the data processing and modelling methods applied, the validation strategies involved, the integration strategies encompassed, and the evolution of prognostic predictive models are discussed. Finally, we discuss challenges and opportunities in this field of cancer research, with great potential impact on the clinical management of patients and, by extension, on the implementation of personalised and precision medicine.

**Abstract:**

Cancer is one of the most detrimental diseases globally. Accordingly, the prognosis prediction of cancer patients has become a field of interest. In this review, we have gathered 43 state-of-the-art scientific papers published in the last 6 years that built cancer prognosis predictive models using multimodal data. We have defined the multimodality of data as four main types: clinical, anatomopathological, molecular, and medical imaging; and we have expanded on the information that each modality provides. The 43 studies were divided into three categories based on the modelling approach taken, and their characteristics were further discussed together with current issues and future trends. Research in this area has evolved from survival analysis through statistical modelling using mainly clinical and anatomopathological data to the prediction of cancer prognosis through a multi-faceted data-driven approach by the integration of complex, multimodal, and high-dimensional data containing multi-omics and medical imaging information and by applying Machine Learning and, more recently, Deep Learning techniques. This review concludes that cancer prognosis predictive multimodal models are capable of better stratifying patients, which can improve clinical management and contribute to the implementation of personalised medicine as well as provide new and valuable knowledge on cancer biology and its progression.

## 1. Introduction

Currently, cancer is one of the biggest public health problems and has a great economic impact on every health system around the world because of its high incidence, prevalence, and mortality. According to a recent review of epidemiological data on cancer, the global cancer burden increased to 19.3 million new cases and 10 million deaths in 2020 [1]. It is estimated that 1 in 5 men and women worldwide will be diagnosed with cancer in their lifetime, and 1 in 8 men and 1 in 11 women will die from this disease.

The GLOBOCAN 2020 database [1], provided by the International Agency for Research on Cancer (IARC), has reported that female breast, lung, and colorectal cancers are the three types of cancer with the highest incidence, whereas the highest mortality rate is attributed to lung, colorectal, liver, stomach, and female breast cancer. The disease is among the top two causes of death before age 70 in almost two-thirds of all countries and its prevalence increases steadily. The estimate is that by 2040 the number of cancer cases will be 28 million and that 16 million people will die from the disease [2].

Due to the high rates of mortality and morbidity, cancer is placing a growing demand on healthcare systems and leading to significant detrimental economic effects. There is evidence that links higher cancer morbidity and mortality with a lower gross domestic product [3]. This challenging context, defined by social, health, and economic factors, requires a holistic approach that integrates prevention, early diagnosis, and adequate medical care to tackle this problem.

Regarding cancer prevention, main prevention and early detection multi-level strategies have aided in reducing cancer incidence [4]. It is estimated that 40% of cancers in Europe could be prevented by educating the population in reducing the exposition to common risk factors as well as by carrying out tests for early detection of premalignancy in high-risk individuals and doing a better follow-up of cancer survivors to prevent cancer recurrence [5].

The cornerstone of cancer diagnosis is the histomorphological study of the tumour performed by pathologists, in which the cancer type and subtype are defined. In the past few decades, a series of molecular techniques such as immunohistochemistry (IHC) and the polymerase chain reaction (PCR) as well as genetic profiling methods such as multiplex real-time PCR and Next Generation Sequencing (NGS) have been developed and optimised to identify genetic aberrations and other relevant molecular biomarkers. The combined use of the conventional histomorphological study and the ancillary tests mentioned above have enabled the pathologists not only to diagnose with greater finesse but also to predict a more accurate clinical outcome through the detection of biomarkers with prognostic value [6].

At the beginning of the 20th century, the therapeutic approach in Medical Oncology was shifted due to the discovery of chemotherapy and its application in the treatment of various tumours. Much more recently, the new paradigm of targeted therapy has prompted the research and development of drugs for specific molecular targets, with the consequent increase in survival and improvement of the quality of life of cancer patients, even leading to complete remission in some cases [7]. In the last few years, the breakthrough caused by omics research has brought new therapeutic approaches for cancer treatment including the identification and validation of genetic alterations with therapeutic value and the design of therapies for advanced tumours. This progress is leading to the implementation of precision medicine [8].

At present, research efforts are focused on the use of multi-omics data to achieve a better understanding of cancer progression and anti-cancer drug sensitivity. Translating genomics and other omics data into clinically effective targeted therapies requires an integrated and multidisciplinary approach that allows for the identification of novel predictive factors or even molecular profiles that reflect cancer resistance as well as its vulnerabilities. Right along this path, new Artificial Intelligence (AI) techniques can contribute to addressing the core issues of this formidable task, including the processing of massive multi-omics data sets and their integration with other types of data, such as clinical or imaging data [9].

Cancer prognosis is the prediction of the evolution of the disease. Knowing the prognosis is key to estimating the probability of cancer progression and life expectancy, which subsequently impacts the clinical management of the patient [10]. Usually, the prognosis is assessed from clinical variables, as happens with the Tumour-Node-Metastasis (TNM) staging system [11], the Nottingham Prognostic Index (NPI) for breast cancer, and the Fédération Internationale de Gynécologie et d’Obstétrique (FIGO) stage for gynaecological tumours. However, there is an increasing trend toward using molecular testing to this end—for example, the Prediction Analysis of Microarray 50 (PAM50) and the Oncotype DX tests provide the risk of recurrence for breast cancer. This additional information has become essential to clinicians when defining a therapeutic strategy and monitoring the evolution of a patient’s condition.

Meanwhile, survival analysis is a hotspot in clinical research. Survival analysis is a subfield of statistics that aims at estimating the time until the occurrence of an event of interest providing the probability of the event occurrence at each time point [12]. In Oncology, this event may be, namely, local recurrence, distant metastasis, or death. Therefore, several concepts related to the survival likelihood of an oncologic patient are commonly used when a prognosis is given. Some of them are: (a) the risk of recurrence (e.g., local recurrence or distant metastasis), which is the likelihood that a treated cancer will reappear; (b) cancer-specific survival (CSS), defined as the period from the diagnosis until death due to a specific type of cancer; (c) progression-free survival (PFS), defined as the period after treatment when the disease, which could not be eliminated, does not progress; (d) disease-free survival (DFS), which is the period after the disease is eliminated when no disease can be detected; and (e) overall survival (OS), defined as the period from diagnosis to death or last follow-up, with no restriction on the cause of death.

Recent studies are focusing on providing better survival estimation based on multimodal data such as clinical, molecular, and image data. The combination of multimodal data may offer a more in-depth description of the underlying characteristics affecting the survival and their interrelationships in contrast to the individual modalities [13]. Two main strategies are being used for such purposes. The first approach is based on the most commonly used traditional techniques for survival analysis, which include the Kaplan–Meier estimator [14], the log-rank tests [15,16], and the Cox Proportional Hazard (CPH) regression [17]. The Kaplan–Meier estimator produces survival curves, the log-rank test is a non-parametric statistical comparison between two groups, whereas the CPH model also estimates survival but it allows other explanatory variables to be considered [18]. A second strategy is an AI-based approach that uses algorithms to build predictive models from prognostic features. Whereas conventional statistical methods are simpler to implement and understand and require little computational power, they fail when it comes to the processing and integration of massive, high-dimensional data. Predictive models created with traditional methods have been proven to perform well with low-dimensional data sets containing clinical (e.g., age, gender, histological grade, stage, etc.) and molecular data (e.g., mutation status of relevant genes, expression of proteins determined by IHC, etc.), but this is not the case when learning from omics data (e.g., genomics, epigenomics, transcriptomics, proteomics, etc.) or image data (e.g., histological images, magnetic resonance images, computed tomography scans). Fortunately, the newest AI techniques can deal with the challenges that this complex and high-dimensional data poses. A wide variety of Machine Learning (ML), especially Deep Learning (DL) algorithms, have been used for this purpose with overall success [10,19,20,21,22,23]. Indeed, in recent years the application of ML techniques to personalised medicine in order to enhance the accuracy of cancer progression and survival prediction has led to an improvement of 20–25% in the prediction of cancer prognosis [24].

Nevertheless, not even ML techniques can overcome many of the biggest limitations of the abovementioned goal; namely, the lack of data to build models as well as independent data sets to externally validate them; the curse of dimensionality; the complex task of integrating massive, multimodal and, many times, sparse data [25], sometimes being necessary a priori or specialist knowledge, or the application of feature engineering techniques to eliminate redundancy within the data set whilst keeping the most informative features; the imbalance in data types, which, if not corrected, will likely result in a biased model; the need of great computational capacity; and the ever-growing importance of building models that are understandable for non-expert audiences [26].

This review aims to present the state-of-the-art on multimodal data integration techniques to improve cancer prognosis. It is intended to give a clear view of the state of the art, targeting both a medical and an IT audience given the multidisciplinary nature of the subject. This paper details the data modalities used; the models and processing methods and the types of integration strategies adopted, culminating with a discussion of how predictive prognostic models have evolved; their current limitations; glimpsing future trends; and outlining the great potential impact of this line of research applied to personalised and precision medicine.

The most recent and relevant scientific publications are presented and analysed, providing the reader with a comprehensive view of the topic. The structure of the present work is as follows. Section 2 explains the implemented methodology for the collection of the selected studies. Section 3 provides some background by describing the data used for training models of cancer prognosis prediction, as well as some highlights of Machine Learning and a brief description of methods for integration of multimodal data. Section 4 presents the results of this review in-depth, pointing out the most common types of data used in the reviewed articles, as well as the approaches for data integration. In addition, the predictive models’ features are described, and the most common techniques for building and validating these models are outlined. Lastly, information on common data sources from which multimodal data sets are obtained is summarised. Section 5 synthesizes the findings of the review and the issues and challenges as well as future expectations in the domain. Finally, Section 6 depicts the conclusions drawn from this state-of-the-art review.

## 2. Methods

A narrative non-systematic review of the literature was carried out to summarise, through an analysis of the temporal progress, the main techniques for predictive modelling applied to cancer prognosis. A search of related scientific literature published in the last 6 years was performed in February 2021 using the Web of Science Core Collection (WoS) and in MEDLINE search engines. Search terms included ‘cancer’; ‘predict’; ‘prognosis’; ‘survival’; ‘machine learning; ‘deep learning’; ‘multi*’ and ‘integrati*’. Studies were first screened by title and abstract, and the full text of those studies that applied molecular, imaging, and clinical data analysis for predicting cancer prognosis were reviewed. Only articles that provided all the information needed to discuss and compare results were retained. Further, a manual review of the references list for the selected articles was conducted to screen for supplementary works of interest.

The inclusion criteria were as follows: (1) articles that integrated multimodal information of at least two of the following types: clinical, anatomopathological, genomics, epigenomics, transcriptomics, proteomics, non-omics molecular, or imaging (histological/radiological) information; aimed to build predictive models of cancer prognosis; (2) the study presented information on the algorithms used or frameworks developed for cancer prognosis; and (3) the article was written in English and published from 2016 to 2021. Abstracts, posters, and reviews were not considered.

Every author of this manuscript independently reviewed all articles, and a consensus on all included studies was reached. For each article, data were extracted regarding (1) authors; (2) year and country of the author group; (3) study design and aims; (4) data source; (5) sample size; (6) input data type and methods used to gather data; (7) use of feature engineering before or during the training of the model; (8) ML algorithms or statistical models used; (9) type of multimodal data integration adopted; (10) internal validation techniques and performance metrics; (11) external validation; (12) dimensionality reduction techniques applied; (13) output variables; and (14) model comparison.

## 3. Background

### 3.1. Multimodal Data

#### 3.1.1. Overview of Multimodal Data

One of the greatest current challenges in biomedical research is to deal with the features derived from large data sets that integrate clinical records, imaging, and high-throughput omics data. In this article, we use the term ‘multimodal data’ or ‘multi-view data’ to refer to a set of data of different features and sample sets, generated from heterogeneous sources that can provide complementary information to support the characterization of a biological sample, an event, or a system, with special application to cancer prognosis. More specifically, a study was considered to involve multimodal data processing if data from at least two of the following categories were used: clinical, anatomopathological, genomics, epigenomics, transcriptomics, proteomics, non-omics molecular, and medical imaging data.

Multimodal data are integrated and used to perform data-driven analyses aimed at facing problems such as feature selection, classification, regression, unsupervised learning, inter-view interactions, and association studies [27]. Through the many articles of this review, multimodal data has been fed to algorithms able to accept different data types to build predictive models on cancer prognosis, addressing mostly binary classification problems. In the following subsections, these types of data are presented.

#### 3.1.2. Clinical Data

This type of data comprises mostly demographic data, general measures of health status, laboratory test results, surgery-related data, pathological data, and therapy-related data.

#### 3.1.3. Molecular Data

In this work, we make a distinction between omics data, understood as massive data obtained with high-throughput techniques, and non-omics molecular data, obtained with traditional techniques that rather focus on a small number of targets. Concerning Omics, we focus on genomics, epigenomics, transcriptomics, and proteomics [28,29,30,31,32,33,34], given that these are the types of omics data used in the reviewed papers. Some types of molecular data found in the reviewed articles do not fit the description of omics data. These non-omics data are mainly: (a) data derived from IHC techniques [35]; and (b) genetic data obtained with PCR techniques [36].

#### 3.1.4. Image Data

A variety of biomedical imaging techniques are used routinely in the management of cancer patients: imaging is an important part of cancer clinical programs since it can provide structural, morphological, metabolic, and functional information [37].

In cancer, clinical images usually include histologic images, in the form of whole slide images (WSIs) of histological samples, as well as radiological images, including magnetic resonance images (MRI), computed tomography (CT) scans, positron emission tomography (PET), and mammographic images.

In this regard, the term ‘radiomics’ is used to refer to the extraction and analysis of high-dimensional quantitative imaging features from medical images obtained with CT, PET, or MRI [38].

### 3.2. Machine Learning

Machine Learning, in its branches of shallow Learning (SL) and Deep Learning (DL) [39], has proven to be a promising area in biomedical research, where it has been applied to a plethora of domains through different techniques and algorithms [40].

DL has attracted much attention for its potential value in different types of real-world applications including key areas of medicine such as medical imaging and genomics [41]. While the design of an SL system requires domain expertise and human engineering to develop feature extractors that extract features from the data to allow learning algorithms to detect patterns, this is not the case for DL methods, whose algorithms contain multiple levels of representation and multiple layers of non-linear processing units, directly taking raw data and building the internal representations needed for recognition.

#### 3.2.1. Model Evaluation and Performance Metrics

Once an SL or DL model is obtained, it is paramount to estimate its performance. The performance analysis of any ML model is usually quantified in terms of standard metrics such as sensitivity (Sn), specificity (Sp), accuracy (Acc), area under the curve (AUC), and mean absolute error (MAE), among others [42].

Internal evaluation processes involve splitting the initially labelled data set into subsets using different approaches such as hold-out, random sampling, cross-validation, or bootstrap [43].

#### 3.2.2. Dimensionality Reduction

It is well known that SL algorithms perform better when the number of variables in a data set is lower than the number of observations [42]. The opposite situation results in the ‘curse of dimensionality’. To overcome this issue and eliminate redundancy, dimensionality reduction techniques are often applied [44]. Dimensionality reduction can be achieved through two different feature engineering techniques: feature selection and feature extraction.

Feature selection approaches are used to find a subset of features that efficiently represents the data by selecting only the relevant and removing the redundant ones. Methods for feature selection can be classified into three main categories: filter, wrapper, and embedded methods [45]. While filter-based methods are independent of the ML model, wrapper approaches are linked to the predictive ML model given that it selects a set of features that improve the model performance. Nevertheless, wrapper methods are often limited in the omics field given the low computing efficiency in those large data sets. Embedded methods also rely on ML models but are less computationally expensive.

On the other hand, feature extraction aims at reducing the number of features by transforming the original high-dimensional data set into a new low-dimensional data set with minimum information loss and a higher discriminating power. Among the most common multi-domain methods used for feature extraction are the Principal Component Analysis (PCA), Kernel PCA, Bayesian PCA, Principal Coordinates Analysis (PCoA), Correspondence Analysis (CA), Independent Component Analysis (ICA), sparse methods, autoencoders, Multidimensional Scaling (MDS), Locally Linear Embedding (LLE), Linear Discriminant Analysis (LDA), and clustering methods [46,47].

##### Multi-Omics Pre-Processing and Dimensionality Reduction

In general, it is accepted that high-dimensional multi-omics data analysis can provide more complete biological information than single-omics data [48]. Nevertheless, multi-omics data analysis poses specific computational challenges such as the curse of dimensionality, data heterogeneity, the existence of missing data, and scalability issues, among others [49]. First, multi-omics data from high-throughput sources are generally heterogeneous and require pre-processing [50]. Among the most common pre-processing steps are normalization, scaling, imputation [51], and outlier detection techniques [52]. Imputation and outlier detection techniques need to be applied to each omics independently before proceeding to data analysis and integration [53]. In addition, the number of multi-omics features is generally greater than the number of biological samples, which leads to the curse of dimensionality and affects the algorithm performance. Dimensionality reduction (DR) techniques such as the abovementioned feature selection and feature extraction methods are broadly used to reduce the dimensional data space.

In particular, the application of DR for effective multi-omics data integration is a field of great interest, and specific approaches are being proposed. In this regard, clustering techniques are broadly extended for their potential to unveil systemic information albeit at the expense of a large computational burden. A recent review of state-of-the-art algorithms for multi-omics clustering applied to cancer research, including similarity-based methods, general dimension reduction, statistical methods, and DL approaches, has highlighted the key aspects that need to be considered in relation to the choice of the clustering approach [54]. Very recently, joint dimensionality reduction (jDR) methods have been presented as an efficient approach for the study of cancer omics, assessing their strengths in predicting survival and extracting new knowledge from biological processes. Due to the vast extent of existing DR methods, we invite the reader to consult the works of [22,55,56] and [54] for more information.

In any case, it is important to emphasize that the chosen DR technique must be consistent with the multimodal data integration technique selected to tackle the problem. Integration techniques will be discussed in the next section.

### 3.3. Data Integration

In recent years, new methods have been introduced to integrate and analyse multimodal data producing new diagnostic and classification biomarkers and enabling the improvement of clinical outcome prediction [22,57].

Today, a myriad of data integration methods is available including supervised and unsupervised learning algorithms. There is no rule of thumb for the pre-hoc selection of a given strategy. The most efficient approach requires empirically testing multiple methods on the available data set [53].

The major challenge lies in the integration of multi-omics information, by nature of very high dimensionality and complexity. Therefore, the following subsection addresses the methodologies applied in multi-omics state-of-the-art studies. These strategies are currently generalised for the integration of medical imaging, clinical, and non-omics data.

#### Multi-Omics Integration

In the last few years, specific approaches for the data integration of different high-dimensional multi-omics data sets have been developed. These strategies can be broadly divided into three categories, depending on the stage at which the integration becomes effective. While the terminology used varies, the two most frequent groupings are those that establish ‘early’, ‘intermediate’, and ‘late’ integration methodologies [56,58,59]; and those that designate them as ‘concatenation-based’, ‘transformation-based’, and ‘model-based’ [53,60]. Although the nomenclature differs, the underlying concepts are similar. In this review, we will describe the approaches according to the first grouping option.

Early integration is based on the concatenation of multi-omics data into a single data set. Once this joint matrix is created, the conventional analysis techniques in the field of single omics can be applied (e.g., clustering) [59]. Commonly, this matrix is used as input to ML-based models—including both SL and DL approaches—capable of finding hidden patterns among variables.

The concatenation of different omics increases the size of the data space at the expense of increasing the number of variables, exacerbating the ‘curse of dimensionality’. Consequently, in most cases where early integration is applied, DR needs to be carried out in order to reduce the number of variables by either applying it to the separate single-omics data sets prior to concatenation or directly onto the concatenated joint matrix. In this latter case, it is ensured that all omics are addressed during the process and potential interactions between omics are considered. Some studies that apply an early integration strategy use autoencoders [61], artificial neural networks (ANN) [62], Mixed Graphical Models (MGM) [63], and Graphical Random Forest [64] to combine the different omics layers in a compressed joint matrix with reduced dimensionality.

Although early integration is easy to implement, it cannot correct imbalance within multi-omics data sets due to heterogeneous sizes in single-omics data sets, which could have a detrimental effect on the predictive models. In addition, there is a potential information loss because early integration does not consider the individual contribution of each individual omics. Despite these potential disadvantages, the use of early integration can provide excellent results in some scenarios.

In intermediate integration, the multiple omics layers are jointly analysed without simple concatenation. In general, intermediate integration often requires prior DR to be more effective. Similarity-based integration (e.g., kernel learning, spectral clustering approaches, graph fusion algorithms), jDR, Non-negative Matrix Factorization (NMF), manifold alignment, autoencoders, and statistical modelling approaches (e.g., Bayesian approaches) are commonly used in intermediate integration schemes [48]. Intermediate integration performs well to unveil underlying biological mechanisms given the complementarity of the information encapsulated in each individual omics.

In late integration, a separate analysis of each omics is performed, and subsequently, the results are integrated to obtain a consensual result or output. This involves the creation of intermediate models for each different omics, and the development of a final joint model that takes as input the output of each of those intermediate models. Mixture model ensemble clustering [65], cluster-of-clusters analysis (CoCA) [66], and Kernel Learning Integrative Clustering (KLIC) [67] are novel techniques used in late integration. Late integration benefits from the possibility of using omics-specific techniques without the challenge of merging heterogeneous data, but at the cost of loss of complementary inter-omics information.

## 4. Results

This paper is the result of a qualitative research study of 43 recent articles related to the prediction of cancer prognosis using multimodal data. Table 1 presents the studies’ characteristics including the reference with the year of publication, the country where the study was conducted, as well as the study design, sample size, cancer type, and the data type used in the multimodal approach. Data types are broadly categorised into clinical, molecular, and image data. Finally, the analytical approach used to develop the predictive model is also shown in the table. We have classified the studies into those applying conventional statistics (n = 6), those based on ML techniques (n = 25), and those that utilise a combination of both (n = 14). Two of the works envisaged two different approaches and therefore are mentioned in two categories.

### 4.1. Sample Size and Cancer Type

The sample size of the reviewed papers ranges from 111 to 11,160 observations, but this does not seem to correlate with the type of cancer, type of study, year when the study was conducted or taken approach, as seen in Table 1. On the other hand, the types of cancer for which predictive models were built are diverse. Breast cancer is the most recurrent one, but the reasons for this are unclear. Several lung carcinoma subtypes appear occasionally, as well as brain tumours such as gliomas and neuroblastoma. Other cancer types studied are cervical cancer, liver carcinoma, and renal carcinoma. Interestingly, seven studies conducted a pan-cancer analysis [76,80,95,97,106,107,109].

### 4.2. Multimodal Data

#### 4.2.1. Clinical Data

In the articles gathered for this review, clinical data is the second most used type of data for building predictive models. Table 2 comprises the subtypes of clinical data, the associated variables, and the reference to the article where they have been used.

Clinical data were used in 28 out of the 43 reviewed articles. Demographic data were used in 23 studies, with age being more used than gender or ethnicity. General measures of health status were considered in 11 studies, with the presence or absence of comorbidities and the body mass index (BMI) being present in 7 and 5 studies, respectively. Laboratory test results data were used in four studies. In all of them, serum metabolite/enzyme levels were used as input to the model. Surgery-related data were used only in one study, and pathological data were considered for 23 predictive models.

Along with demographic information, the pathologic data were the most frequently used clinical data, especially the size of the primary tumour (n = 8), the histologic grade (n = 14), and the stage (n = 12). Finally, therapy-related data were used in 12 studies, being the use of targeted therapy and radiotherapy the most used variables in this category (n = 7), followed by chemotherapy (n = 6).

#### 4.2.2. Molecular Data

Molecular data were used in 39 out of the 43 selected studies, which makes this modality the most used for training cancer prognosis predictive models within the corpus of reviewed articles. Genomics data, including germinal variants, somatic point mutations (e.g., SNVs, indels), mutational status of genes, CNAs, copy number burden (CNB), and tumour mutation burden (TMB) were used as model inputs in 20 studies [76,77,78,79,83,85,88,89,90,92,93,96,97,98,100,103,106,107,108,110]. CNAs were the most used mutations (n = 17). All studies that considered genomics as input data built predictive models based on ML or based on a mixed (conventional statistics and ML) approach.

Epigenomics data, and more specifically DNA methylation data acquired by DNA methylation arrays bisulphite sequencing, was used in 15 studies [61,76,77,85,90,91,92,93,94,95,106,107,108,109,110]. Again, epigenomics data were used to train ML models as well as models that mixed ML and conventional statistical methods.

Transcriptomics data obtained by RNA-Seq [111] or RNA microarrays [112] were considered in 30 studies [61,68,69,76,77,78,79,80,83,85,88,89,90,91,92,93,94,95,96,97,100,102,103,104,105,106,107,108,109,110], with mRNA levels, miRNA levels, and gene expression profiles used as variables. Only two articles [68,69] from the conventional statistics category used mRNA levels for developing predictive models.

Proteomics data were used only in four studies, where protein expression levels were used as input models of ML or mixed models [77,91,92,97].

Table 3 summarises the type of omics data used as input in the selected studies, the methods used to obtain this data, the variables containing the information, and the reference to the papers that use these variables as input for training their models.

Table 4 details molecular data type, the technique used to gather the information in several variables and studies that have compiled this information and used it to enrich their models. IHC data were used for developing models in 10 studies, being the presence/absence of proteins in tumour tissue the most broadly considered (n = 8). Genetic data obtained with PCR techniques were more rarely consumed (n = 4), but it was used to build all three types of predictive models (statistical-based, ML-based, and mixed approaches).

#### 4.2.3. Image Data

Image data (image segmentation and hand-crafted features) were used in 13 of the reviewed articles (Table 5). Eleven studies used histological images and three studies used CT or MRI as input information for machine learning or mixed models. Quantitative image features (n = 6) or regions of interests from WSIs and CTs (n = 7) were used. Two out of the thirteen studies used these data to build predictive models using conventional statistics, four used them with ML techniques, and seven were used for creating mixed models.

### 4.3. Data Integration

Of the manuscripts analysed in this review, the studies of [61,68,70,71,72,73,74,75,78,81,82,84,86,91,97,98,99,106,110] employ an early integration strategy (n = 19). Among the manuscripts discussed in this review, the articles by [69,76,79,80,87,88,89,90,92,93,95,96,100,102,103,104,105,107,108] use an intermediate integration strategy (n = 19). The study of [85] applies both early and intermediate integration of multimodal data.

The studies by [77,83,94,101,109] addressed a late integration strategy (n = 5).

### 4.4. Predictive Models for Cancer Prognosis Prediction

For the sake of simplicity, we have sorted the reviewed articles according to the type of model used. In the selected articles, we may find studies that used a conventional survival analysis, ML, or a mixed approach. In each case, we have identified the type of multimodal data, the statistical or ML methods employed, the validation strategy and the clinical outcome associated with cancer prognosis predicted by the described system.

Out of the 43 studies, 6 studies addressed the model using a conventional statistics approach [68,69,70,71,72,73] and 25 developed ML-based models [61,73,74,75,76,77,78,79,80,81,82,83,84,85,86,87,88,89,90,91,92,93,94,95,96]. In the remaining 14 studies, a combination of both approaches was used [97,98,99,100,101,102,103,104,105,106,107,108,109,110]. One study was counted on both conventional and ML categories since it included ML and statistical models built independently [73]. Similarly, the study [95] was counted in the ML and mixed approaches category.

#### 4.4.1. Conventional Survival Analysis

A total of six articles fall under this category, where we have brought together all the predictive models built using traditional statistical methods. Table 6 describes several characteristics related to how cancer prognosis predictive models are built, such as the sample size of the data set, the application of dimensionality reduction techniques, the statistical methods and validation techniques used, the metrics of performance, the output of the model, and whether the model is externally validated and/or compared to others.

Four studies used the CPH regression model [70,71,72,73], a semi-parametric model able to handle right-censored data whose output is the Hazard Ratio (HR) and that is arguably the most used technique for survival analysis in the medical field [12]. A Lasso–Cox approach [69] and supervised Principal Components regression (superPC) [68] were used in one article.

Dimensionality reduction techniques were used in 4 of the studies [68,69,70,71]. Sequential backwards elimination and feature selection using the HR value was used in [70,71], respectively. Univariate survival analysis and gene co-expression network analysis (GCNA) were carried out from raw transcriptomics data to cluster genes into co-expressed modules that are later summarised as an eigengene using the lmQCM method used in [69]. Finally, sparse partial correlation estimation (SPACE) was applied both to image features and gene expression data for feature selection in [68].

Regarding validation techniques, 10-fold CV was applied in three studies [68,69,73], all of them with less than 1000 patients. Bootstrapping was used in [72], which counted 9182 patients. Finally, two studies involving 5738 and 2186 patients did not apply any validation method [70,71]. Moreover, only two studies provided external validation of their models on independent data sets. The Waikato data sets and the NTBCS, BCOS, and POSH data sets were used in [70,72], respectively.

The performance metrics used to test the robustness and efficacy of the developed methods were heterogeneous. Two studies reported log-rank tests *p*-values [68,69], two presented C-index values [72,73], and one study reported the area under the Receiving Operating Characteristics Curve (AUC) [70]. Median Absolute Error (MAE) and the HR were calculated in two studies [68,73].

This heterogeneity was also shown in the output variable provided by the statistical model developed. Overall survival (OS) was only used in two studies [72,73]. The rest of the studies used very different output variables: HR; a binary classification between high-risk and low-risk patients; a risk index of death, which correlates with survival; risk of death at 10-years; the Lung-molGPA score, correlated with OS; a survival risk index; progression-free survival (PFS); and binary classification of patients according to survival (alive/dead) and metastasis (yes/no).

Finally, a quantitative model comparison with other reported models was addressed in three studies [70,72,73].

#### 4.4.2. Machine Learning-Based Approaches

This category includes the articles whose predictive models are built using exclusively ML algorithms, making a total of 25 articles. Table 7 summarises the key information on these studies.

Ten studies applied DL [61,73,80,82,83,91,92,94,95,96] while the remaining fifteen used SL. The SL models employed were very diverse. Eight studies used algorithms based on decision trees including Bagged Decision Trees (BDT) [74], Random Forest (RF) [79,84,85], Random Survival Forest (RSF) [87], Extreme Gradient Boosting (XGBoost) [78], gradient boosting [79], and AdaBag [84]. Two studies trained K-means clustering classifiers [75,78], and three evaluated a simple Multiple Kernel Learning (MKL) classifier either with Gaussian Kernel [77] and based on SVM [81,88]. Seven studies built predictive models based on Support Vector Machines (SVM) [75,78,79,84,85,86] and one presented a Multi-Omic Kernel (MOK)-based classifier [76]. Four studies applied Artificial Neural Networks [78,79,86,90,93] and some other works considered variations of these, such as a Probabilistic Neural Network (PNN) [75], a Multilayer Perceptron (MP) [75], a Radial Basis Function Neural Network (RBNN) [75], or even an ensemble of Convolutional Neural Networks (CNNs) and RF [89]. Other approaches were based on a Naïve Bayes classifier [84], a Logistic Regression (LR) classifier [85], or an L2 Regularised Regression classifier [85]. Finally, an evolutionary algorithm, a Gene Expression Programming algorithm, was applied in [75].

Dimensionality reduction approaches were used in 20 studies. Feature selection was used based on different methods: Particle Swarm Optimisation (PSO) for categorical features [74], univariate CPH regression analysis [61,78], logistic regression [85,86], minimal Redundancy Maximum Relevance (mRMR) [83,88,89,94,96], the log-rank test [87], or a variance-based feature extraction approach [90].

Feature extraction was applied in several other studies, using kernel-based methods [76,77], autoencoders [61,78,80,85,91], K-means clustering [79], Principal Component Analysis (PCA) [90], Neighbourhood Component Analysis (NCA) [92], non-linear functions [93], or deep embedded features [95].

Several CV schemes (3-fold, 4-fold, 5-fold, 10-fold, and Monte Carlo) were utilised in 18 studies [61,73,74,75,76,77,78,79,81,83,85,86,88,89,90,92,93,94,96]. Hold-out, bagging, and bootstrapping were used in five [74,80,84,85,94], two [74,87], and one studies [82], respectively. Four studies validated the models using multiple validation strategies. Nine studies provided external validation of their models on independent data sets [61,76,78,82,83,87,89,91,92].

The heterogeneous scenario described previously in terms of the diversity of performance metrics implemented is mirrored for ML models. The most commonly used metric was the *Acc*, accounting for fourteen studies [74,75,77,79,81,83,84,85,86,89,92,94,96], followed by the AUC, used in twelve studies [74,75,77,78,81,83,85,86,87,88,89,92]. The C-index followed, being used in 11 studies [61,73,76,77,80,81,82,85,90,93,95]. *Sp* and *Sn* were estimated to measure model performance in eight studies [74,75,81,83,84,86,89,92]. In five studies, precision was estimated [74,77,83,89,96]. Matthew’s correlation coefficient (MCC) [74,77,83,89,92] and positive/negative likelihood ratios [74,84] were used in five and two studies, respectively; F-score [74,81,92] was used in three studies and ROC curve [79,83] in two studies. Barely, the log-rank *p*-value for the CPH regression model [61,91] and the Brier Score (BS) as the error of the model [61], stability and calibration slope [79], predictive values [84], Cohen’s kappa coefficient (κ) [74,92], Diagnostic Odds Ratio (DOR) and Discriminant Power (DP) [74], mean absolute error (MAE) [73], False Positive Rate (FPR) [92], Integrated Brier Score (IBS) [85,95], and Silhouette score [91] were also estimated.

There was a modicum of homogeneity in the ML output variable. Patient risk dichotomization followed OS, being used in seven studies [76,78,81,82,87,92,93]. Overall survival (OS) was primarily used appearing as model output in seven studies [73,75,78,80,85,87,95].

Dichotomization of patients into short/long-term survivors was done in six papers [77,83,88,89,94,96]. Binary recurrence or risk of recurrence [74,84,86] were evaluated as prognosis indexes in three studies. Evaluated recurrence was either loco-regional [74,86] or systemic (metastatic) [84,86].

DFS was used in two articles [82,85]. A binary classification according to survival was used in two studies [79,84]. Finally, an ad-hoc survival risk label [61], predicted overall prognostic score [76], and progression-free disease (PFS) [73] were used in one study each.

To end, a model comparison with models published in the scientific literature was faced in 19 studies [61,73,74,75,76,77,78,79,82,83,84,85,88,89,92,93,94,95,96].

#### 4.4.3. Mixed Approaches

The scientific articles gathered in this section used a combination of ML algorithms and statistical models to predict the clinical outcome of cancer patients from multimodal data. In total, 14 papers are discussed (Table 8).

Twelve studies applied DL [97,98,99,100,101,103,104,105,106,107,109,110] and two used SL [102,108]. Most DL approaches combined DL techniques and CPH as output layer (n = 10), although [109] built an ensemble of DL and SVM models. In articles dealing with SL, several ML algorithms were used but also combined with CPH or Elastic net-CPH.

Eleven out of the fourteen studies compared the resulting model performance with other models [97,98,99,100,101,102,104,106,107,108,109]. Ten of the studies applied dimensionality reduction techniques [100,102,103,104,105,106,107,108,109,110].

All studies except [101,104,106,107] used CV methods with different schemes (ten, five, three, fifteen, Leave-One-Out, Monte Carlo). Bootstrapping [98] and hold-out [98,107] were also used. The validation approach was not detailed in [101,104,106].

Six articles detailed the use of external data sets for model validation [98,101,106,108,109,110]. Performance metrics were more homogeneous than in conventional or ML models. C-index was primarily used in 12 studies [97,98,99,100,101,102,103,104,105,106,108,109]. Log-rank test *p*-value was estimated in three studies [100,101,109]. AUC [101,110] and accuracy [101], BS [102], integrated Brier Score (IBS) [107], and time-dependent C-index (Ctd) [107] were more scarcely used.

The main model output was the high/low risk classification [100,101,102,104,105,106,108,110]. In [103], the patients were dichotomised into short or long-term survivors. The HR [98,99,100] was used in four studies. The risk of death was evaluated in two studies [97,102]. Interestingly, the conditional survival probability in a time span of 30 years was the output of the model presented in [107]. An individual’s recurrence risk index [101] and the patient’s risk of death [109] were also proposed as prognostic indexes.

### 4.5. Data Sources

This section summarises the sources from which data were obtained to build predictive models across the reviewed papers, distinguishing between public repositories and institutional databases, as shown in Table 9. Thirty-three out of the forty-three included articles used data from public repositories, while eleven studies built their models using data from institutional (private) databases.

## 5. Discussion

This paper provides the reader with overall knowledge on the integration of clinical, molecular, and image data with the objective of predicting the clinical outcome of cancer, mainly as recurrence, progression, or death. To that end, the information from 43 state-of-the-art scientific papers has been broken down throughout this article.

Throughout this review, we noticed that three major approaches have been adopted to infer prognosis: (1) the use of multivariate statistical methods; (2) the application of ML algorithms; and (3) the combination of ML methods and statistical tests to build predictive models. It seems that the approach taken has evolved with time. Statistical methods were more prominently used in earlier years, whilst ML-based methods replaced them over time. Mixed approaches that combine ML algorithms and conventional survival modelling were the favourites for quite some time. However, in 2020 and 2021 a number of predictive models were published, and those showed some of the best performances to date—they are based solely on DL architectures or consist of frameworks where DL networks have a special relevance [61,73,80,82,83,91,92,94,95,96], indicating that this approach in predictive modelling is gaining traction in the task of cancer outcome.

Conventional survival modelling techniques are known to work well with low-dimensional data sets, as those containing clinical and non-omics molecular information. However, over the years, the advent and cheapening of high-throughput molecular techniques have generated massive and multi-view data that cannot be handled in the same way. Here comes into play Machine Learning, which not only can deal with bigger data sets, but is also able to model the nonlinear association between variables [12]. Thus, Machine Learning techniques have been adopted to learn from imaging and omics information along with the abovementioned data. In more recent years, Deep Learning, a subfield within Machine Learning, has proven to be a better option to tackle this problem; given that it does not require prior feature engineering, its flexible structure allows for better integration of multimodal data and ultimately does a better job at leveraging the interactions between the different modalities [10]. Several publications present predictive models that result from the combination of DNN and CPH regression as the last layer of the DL architecture. This strategy aims to provide the best of both worlds since ML algorithms struggle to deal with censored data in survival analysis [12]. However, it seems like DL-based models have become sophisticated enough to take in and process censored survival data, since many of the more recently published papers present DL-based frameworks able to manage without CPH models to estimate survival time or hazard of event, as mentioned above.

An essential concern in this matter is ascertaining whether the integration of multimodal data improves cancer prognosis prediction. Although it has been widely accepted that multimodal data provides a more complete picture of the topic of research, the existing algorithms for DR and predictive modelling are not always capable of achieving an optimal integration of multimodal data or, on some occasions, some modalities are not as relevant as expected. To this aim, some papers—usually those whose models were fed with a variety of data types—devote a section to the training and comparison of models using different combinations of one or several data modalities [61,68,69,77,80,83,87,97,99,101,102]. In most cases, it is proven that the integration of multimodal data increases the performance values, especially when incorporating multi-omics data.

Considering the boom in omics techniques, the reader might think that this data modality has become central in any experiments focused on the biology of human diseases, but clinical and non-omics molecular data continue to provide valuable information. For example, in [98] IHC data and the mutational status of four genes relevant to the disease, along with gene expression data, are used to predict survival. This multimodal data set is complementary and non-redundant. Furthermore, some upsides of using non-omics molecular data it is low cost and easy to obtain in comparison to omics data, which requires lengthy lab work, data quality control and data pre-processing. Another example can be found in [100], which utilises genomics, transcriptomics, clinical, and IHC data; IHC data indicates the presence or absence of two important protein receptors in breast cancer cells, the oestrogen receptor and the progesterone receptor, which not only have prognostic value by themselves but are also targets of hormone therapy. Again, this IHC information complements the omics data by providing additional information on the status of certain proteins known to be involved to some degree in the progression of cancer and, therefore, in the survival of the patients.

A consequence of these experiments is that new biological information can be drawn. Indeed, many predictive models were not only developed to classify patients according to their risk, but also to explore features or whole data modalities with a high impact on survival prediction. Again, the easiest way of doing so is by sequentially adding or excluding information, re-training the model, and examining the changes in the performance metrics. For instance, Zhu et al. [76] present a survival pan-cancer study that uncovers the most relevant features in prognosis prediction for 14 types of cancer. Surprisingly, it is shown that the molecular profiles obtained from multi-omics data contribute with varying degrees to prognosis prediction depending on the cancer type. The work of Zhao et al. [79] reveals that one cluster of patients highly correlated to increased mortality is defined by the overexpression of 11 genes, some of which were unknown to be linked to cancer. On another hand, Mobadersany et al. [99] is one of the few publications found to integrate image and genetic information for prognosis prediction. In this publication, heatmaps are generated to show the parts of H&E digitised slides that correlate with poor prognosis. Some histological characteristics already known to be associated with it are highlighted, such as microvascular proliferation, whereas low-density infiltrate in the cortex is revealed as a new trait associated with poor prognosis. Finally, Baek and Lee [85] utilise for the first time the cellular prevalence, along with multi-omics and clinical data, for cancer recurrence and survival prediction. The cellular prevalence feature allows identifying new candidate genes whose mutations have a high impact in the early stages of cancer development.

It is worth mentioning that in the vast majority of the articles, clinical variables are considered as input data for the predictive models. In particular, demographic data and pathological data are among the most used in the articles reviewed, evidencing the importance of this type of information for the estimation of cancer prognosis and the explainability of the models. This is highly relevant since, despite the availability of modern sequencing and medical image acquisition techniques, clinical data are still involved in clinical prognostic models providing significant and valuable information, with the advantage that such data are currently easier to acquire, less expensive, and part of the clinical routine. All these findings, and many others, provide a valuable insight that sheds light on the biology of cancer.

Nevertheless, several problems and limitations arise when tackling the task at hand. The most predominant ones are listed below, and potential solutions are pointed out.

I. Lack of data. Although efforts have been made to extensively collect and provide the scientific community with varied information on cancer (as discussed in Section 4.4), the amount of data is still not enough. The cancer-related data sets found in this review contain hundreds to thousands of observations, but are not as large as data sets from other areas (i.e., finances) that usually contain tens of thousands of observations [113]. According to the curse of dimensionality phenomena, the amount of data required to develop models that ensure statistically reliable results grows exponentially with the dimensionality. Therefore, survival predictive models would improve not only by increasing the sample size but also the follow-up time of patients.
II. Only a few multimodal data sets are publicly available. Access to most existing multimodal data is reserved for the hospitals or research centres that own the data. A change in the data privacy legislation and ensuring the privacy of sensitive medical data by computing on encrypted data is paramount to promoting predictive analysis of private databases [114].
III. Heterogeneity in data. Heterogeneity is present at many levels. Firstly, the data sets gather information from patients of different demographics, types of cancer, and treatments. Although having a representative sample of a population is key to training models with good generalizability, this adds heterogeneity that must be handled properly, especially when it supposes an imbalance in the number of patients of different classes or characteristics (i.e., the information of patients of a rare cancer subtype will likely not be captured by the algorithm). Secondly, the multimodality of data considered in this review inherently entails heterogeneity, and the data sets from the reviewed papers seldom gather all the four main types of data discussed in Section 3. Thirdly, whenever possible, models should be able to deal with missing data. Even within a data set, many patients will contain missing data, as not all of them undergo the same tests and follow-up period. Fourthly, the experimental techniques used to gather the anatomopathological, non-omics, and omics data are extremely varied, which influences the amount and quality of data, the format of the data itself, and the pre-processing needed.
IV. Data integration. The availability of multi-omics data has brought about a breakthrough in information analysis techniques. The complexity of these techniques and the difficulty of choosing the optimal ones for each case requires the collaborative effort of multidisciplinary teams that include experts in the field of Data Analysis, Statistics, and Machine Learning who can guide and support the data treatment and development of robust and generalisable models.
V. Lack of external validation. Another limitation is the lack of independent data sets to externally validate the generated models. External validation is paramount to detect potential issues as bias or overfitting and demonstrate the generalisation capability of models [24]. Many studies do not validate the predictive models with independent data sets, although there is an increasing trend to do so. Fortunately, the accessibility to cancer-related data sets grows bigger every day.
VI. Most studies are single-institution and retrospective, while multi-centric and prospective studies are very scarce. Multi-centric studies often result in data sets with a bigger number of observations, and the data collected tends to more accurately reflect the variety of features displayed by the subjects of a population. Additionally, new data gathered in prospective studies could be useful in the validation of the predictive models trained with the initial data.
VII. Difficulty in comparing state-of-the-art models. Experimental replicability and reproducibility are pivotal topics in ML. There is no unified performance measure used in the reviewed articles, which makes a fair quantitative comparison almost impossible. Further efforts should be made to establish common practices that should be evaded to fairly compare results with the state-of-the-art [115].
VIII. The ‘black box’ problem. Most ML algorithms operate in such a complex manner that understanding how information is processed becomes challenging, thus turning the trained models into opaque systems [116]. Naturally, non-expert audiences cannot completely trust them with tasks as important as the management of patients. However, the rise of Explainable Artificial Intelligence (XAI) is contributing to solving this problem and paving the way for the application of ML models in clinical practice [26].

While it provides a review of recent literature, this review has limitations, as it is a narrative non-systematic. As a result, the evidence presented does not encompass an exhaustive synthesis. Despite this, the strength of a narrative review is that it builds on a research area by offering a summation [117]. In addition, we only included studies published in English to increase feasibility, which may have excluded relevant studies published in other languages; however, this is an unlikely source of bias [118].

## 6. Conclusions

To the best of our knowledge, this is the first review focused on the integration of multimodal data comprising clinical, anatomopathological, omics, molecular non-omics, and medical imaging data in order to predict cancer prognosis.

Cancer poses a threat that requires an attack from all angles. Research on cancer prognosis is an open front in which much progress has been made. Cancer is a heterogeneous and complex disease whose origin and evolution are governed by multiple genetic and environmental factors, many of which remain unknown. The evolution of current clinical environments is favouring the collection of multimodal data. It is also our belief that only by integrating data from as many modalities as possible could cancer prognosis prediction be made in the most accurate way, given the incredible complexity of this disease. In fact, this review is proof that the integration of multimodal, multi-view data provides a more complete and holistic approach to cancer outcome prediction.

On another hand, this review suggests that the development of predictive models with clinically useful reliability is evolving. The use of ML techniques has shifted conventional statistical approaches, making possible the handling of complex and massive multimodal data sets using a multi-faceted data-driven approach and successfully achieving a better identification of subgroups of patients of different risk. This methodological approach not only has the potential to improve clinical management and contribute to the implementation of personalised medicine, but also to generate new knowledge on cancer biology and the processes leading to its progression.

Thus, it is not far-fetched to expect a breakthrough in this exciting, emerging field in the coming years that will revolutionise cancer research as we know it.

## Figures and Tables

**Table 1:** Description of the characteristics of the selected studies.

First Author & Reference	Year	Country	Study Design ^1^	Sample Size ^2^	Cancer Type	Clinical Data		Molecular Data					Image Data	Predictive Analytics
						AP	Other	Omics				Non-Omics		
								G	E	T	P			
Zhu [68]	2016	USA	RCS	111 patients	LUAD					✔			✔	Conventional Statistics
Cheng [69]	2017	USA, China	RCS	410 patients	ccRCC					✔			✔	
Dos Reis [70]	2017	UK	MC RCS	5738 patients	Breast cancer	✔	✔							
Sperduto [71]	2017	USA	MC RCS	2186 patients	NSCLC	✔	✔					✔		
Elwood [72]	2018	New Zealand	MC PCS	9182 patients	Breast cancer	✔	✔					✔		
Matsuo [73]	2019	USA	RCS	768 patients	Cervical cancer	✔	✔							
Mohebian [74]	2017	Iran, Spain	SI RCS	579 patients	Breast cancer	✔	✔					✔		Machine Learning
Obrzut [75]	2017	Poland	SI RCS	102 patients	Cervical cancer	✔	✔					✔		
Zhu [76]	2017	USA	RCS	3382 samples	14 types of cancer	✔	✔	✔	✔	✔				
Chaudhary [61]	2018	USA	RCS	360 patients	Hepatocellular carcinoma				✔	✔				
Sun [77]	2018	China	RCS	578 patients	Breast cancer		✔	✔	✔	✔	✔		✔	
Zhang [78]	2018	USA, China	RCS	380 samples	Neuroblastoma			✔		✔				
Zhao [79]	2018	USA	MC PCS	1874 patients	Breast cancer	✔	✔	✔		✔		✔		
Cheerla [80]	2019	USA	MC RCS	11,160 patients	20 types of cancer	✔	✔			✔			✔	
Ferroni [81]	2019	Italy	SI PCS	454 patients	Breast cancer	✔	✔					✔		
Jing [82]	2019	China	MC RCS	4630 patients	Nasopharyngeal carcinoma	✔	✔							
Matsuo [73]	2019	USA	RCS	768 patients	Cervical cancer	✔	✔							
Sun [83]	2019	China	RCS	1980 patients	Breast cancer	✔	✔	✔		✔				
Tapak [84]	2019	Iran	RCS	550 patients	Breast cancer	✔	✔					✔		
Baek [85]	2020	South Korea	RCS	177 patients	Pancreatic adenocarcinoma	✔	✔	✔	✔	✔				
Boeri [86]	2020	Italy	RCS	610 patients	Breast cancer	✔	✔					✔		Machine Learning
Choi [87]	2020	South Korea	MC CS-RCS	205 patients	Glioblastoma multiforme	✔	✔					✔	✔	
Zhang [88]	2020	China	RCS	251 patients	Glioblastoma multiforme			✔		✔			✔	
Arya [89]	2020	India	RCS	1980 patients	Breast cancer	✔	✔	✔		✔		✔		
Tong [90]	2020	USA	RCS	~1000 patients	Breast cancer			✔	✔	✔				
Owens [91]	2021	UK	RCS	352 patients	Hepatocellular carcinoma					✔	✔			
Malik [92]	2021	India	RCS	532 patients	Breast cancer	✔	✔	✔	✔	✔	✔			
Zhao [93]	2021	China	RCS	474 patients	Low-Grade Glioma			✔	✔	✔				
Hassanzadeh [94]	2021	USA	RCS	836 patients	3 types of cancer				✔	✔				
Zhang [95]	2021	UK	RCS	131 patients	35 types of cancer				✔	✔				
Chharia [96]	2021	India	RCS	1980 patients	Breast cancer		✔	✔		✔				
Yousefi [97]	2017	USA	RCS	3323 patients	5 types of cancer	✔	✔	✔		✔	✔			Mixed Approach
Katzman [98]	2018	USA	RCS	1980 patients	Breast cancer		✔	✔				✔		
Mobadersany [99]	2018	USA	RCS	769 patients	Gliomas	✔						✔	✔	
Huang [100]	2019	USA, China	RCS	583 patients	Breast cancer		✔	✔		✔		✔		
Wang [101]	2019	China	MC RCS	245 patients	HGSOC	✔							✔	
Shao [102]	2020	China	RCS	1324 patients	LUSC, breast cancer, LIHC					✔			✔	
Chen [103]	2020	USA	RCS	1186 patients	Glioma and ccRCC			✔		✔			✔	
Hao [104]	2020	USA	RCS	447 patients	Glioblastoma multiforme		✔			✔			✔	
Ning [105]	2020	Germany	RCS	209 patients	ccRCC	✔				✔			✔	
Zhang [95]	2021	China	RCS	454 patients	Bladder cancer			✔	✔	✔				
Chai [106]	2021	China	RCS	5032 patients	15 types of cancer			✔	✔	✔				
Vale-Silva [107]	2021	Germany	RCS	11,081 patients	33 types of cancer	✔	✔	✔	✔	✔			✔	
Wang [108]	2021	China	RCS	Not specified	7 types of cancer			✔	✔	✔				
Poirion [109]	2021	USA	RCS	10,000 samples	32 types of cancer				✔	✔				

Abbreviations: AP, Anatomopathological; G, Genomics; E, Epigenomics; T, Transcriptomics; P, Proteomics; RCS, retrospective cohort study; MC, multi-centric; PCS, prospective cohort study; SI, single-institution; CS, cross-sectional; LUAD, Lung Adenocarcinoma; ccRCC, Clear Cell Renal Cell Carcinoma; NSCLC, Non-Small-Cell Lung Cancer; HGSOC, High-grade serous ovarian cancer; LUSC, Lung Squamous Cell Carcinoma; LIHC, Liver Hepatocellular Carcinoma. ^1^ MC and SI terms were assigned when available. ^2^ Number of patients used to build, train, and internally validate the predictive model.

**Table 2:** Clinical variables by subtypes and bibliographical reference to the article in which they appear.

Subtype of Clinical Data	Variables	Used by
Demographic data	Age at diagnosis	[70,71,72,73,74,75,76,77,79,80,81,82,83,84,85,87,89,92,97,98,100,104,107]
	Gender	[76,80,82,85,87,89,92,97,98,104,107]
	Ethnicity	[72,73,80,89,98,107]
General measures of health status	BMI	[73,75,81,82,98]
	Temperature	[98]
	Respiration rate	[98]
	Systolic and diastolic blood pressure	[73,98]
	Heart rate	[73,98]
	Menopausal status	[79,81,89,97]
	Lifestyle (e.g., smoking habit)	[85]
	Prior malignancies	[107]
	Presence/absence of comorbidities (e.g., hypercholesterolemia, hypertension, diabetes mellitus, synchronous malignancies, etc.)	[73,75,81,85,89,98,107]
	Number of comorbidities	[98]
	Risk factors (e.g., high sensitivity to C reactive protein, etc.)	[76,82,98]
Laboratory test results data	Blood cells count (e.g., leukocytes, platelets)	[73,98]
	Haemoglobin level	[73,82]
	Serum metabolites/enzymes level (e.g., sugar, urea, creatinine, bicarbonate, albumin, lactate dehydrogenase, etc.)	[73,81,82,98]
Surgery-related data	Surgery time	[75]
	Median blood lost	[75]
	Presence of intraoperative complications	[75]
	Type of complications	[75]
	Length of hospital stay	[75]
Pathological data	Mode of detection (clinical or screening)	[70]
	Cancer type (primary site)	[80,85,107]
	Cellularity of tumour content	[79]
	Degree of abnormality of cancer cells	[79]
	Primary tumour laterality	[79]
	Primary tumour size	[70,72,74,75,79,83,86,89]
	Presence/absence of multifocal tumours	[86]
	Surgery status	[73,79]
	Type of surgery	[74,84,89]
	Resection extent	[87]
	Parametrial involvement (in cervical cancer)	[75]
	Skin or chest wall invasion (in breast cancer)	[86]
	Lymph node status	[75]
	Number of positive lymph nodes	[70,72,83,86,89,92,97]
	Lymph node involvement ratio	[74,75]
	Lymph-vascular space invasion	[72,75]
	Deep stromal invasion	[75]
	Histologic type and subtype	[72,73,75,76,84,92,97]
	Histological grade	[70,75,76,80,81,83,84,85,86,87,89,92,99,105]
	T Stage	[82]
	N Stage	[82,92]
	M Stage	[86,92,105,107]
	Stage (e.g., pTNM, NPI, FIGO staging system)	[73,75,76,79,81,84,85,92,97,101,105,107]
	Number of brain metastases	[71]
	Presence/absence of distant metastasis at diagnosis	[72]
Therapy-related data	Prior treatment	[89,107]
	Radiotherapy (yes/no)	[73,75,85,89,97,98,107]
	Chemotherapy (yes/no)	[70,73,79,89,98,107]
	Targeted therapy (yes/no) (e.g., hormonal therapy, anti-HER2 therapy, etc.)	[70,74,79,86,89,98,107]
	Response to chemotherapy (complete/partial/none)	[86]
	Karnofsky Performance Status (KPS)	[71]

Abbreviations: BMI, Body Mass Index; pTNM, pathological Tumour-Node-Metastasis staging system for cancers of the American Joint Committee on Cancer (AJCC); NPI, Nottingham Prognostic Index; FIGO, Fédération Internationale de Gynécologie et d’Obstétrique.

**Table 3:** Summary of types of omics data, methods used to obtain them according to the scientific paper, and variables used to build predictive models along with the bibliographical reference.

Type of Omics Data	Methods	Variables	Used by
Genomics	WGS WES Targeted sequencing DNA microarrays	Germinal variants	[76]
		Somatic point mutations (e.g., SNVs, indels)	[76,85,89,90,92,93,96,103,106,107,108,110]
		Mutational status of genes	[79,90,98,103]
		CNAs	[76,77,78,79,83,88,89,90,92,93,96,97,103,106,107,108,110]
		CNB	[100]
		TMB	[100]
Epigenomics	DNA methylation arrays Bisulphite sequencing	DNA methylation data	[61,76,77,85,90,91,92,93,94,95,106,107,108,109,110]
Transcriptomics	RNA-Seq RNA microarrays	mRNA levels	[61,68,69,76,77,78,79,80,85,88,89,90,91,92,94,96,97,100,102,103,107,108,109]
		miRNA levels	[76,80,85,90,91,92,94,100,107,108,109]
		Gene expression profiles	[83,93,95,104,105,106,110]
Proteomics	RPPA	Protein expression levels	[77,91,92,97]

Abbreviations: WGS, Whole Genome Sequencing; WES, Whole Exome Sequencing; RPPA, Reverse-Phase Protein Arrays; CNAs, Copy Number Aberrations; SNVs, Single-Nucleotide Variants; CNB, Copy Number Burden; TMB, Tumour Mutation Burden. The Copy Number Burden is a measure of the copy number alteration level within a genome in proportion to the genome length. The Tumour Mutation Burden (TMB) represents the number of somatic mutations per megabase of interrogated genomic sequence. Both are used as predictive biomarkers in cancer.

**Table 4:** Summary of non-omics data that appear in the reviewed papers. Methods used to obtain them and the variables containing the pertinent information to build the models are listed along with the bibliographical reference.

Type of Molecular Data	Methods	Variables	Used by
IHC data	Immuno- histochemical staining	Presence/absence of proteins in tumour tissue (e.g., ER, PR, Ki-67)	[72,74,75,81,84,89,98,100]
		Percentage of protein expression in tumour tissue (e.g., ER, Ki-67, etc.)	[86]
		Over-expression of proteins in tumour tissue (e.g., HER-2)	[79]
Genetic data	PCR-based methods	The molecular subtype of cancer (luminal A, luminal B, HER-2 positive luminal B, non-luminal HER-2 positive, triple-negative)	[74]
		Somatic point mutations (e.g., *IDH* R132H mutation)	[87]
		Mutational status of genes	[71,99]

Abbreviations: IHC, Immunohistochemistry; PCR, Polymerase Chain Reaction; ER, estrogen receptors; PR, progesterone receptors; HER-2, Human Epidermal Growth Factor receptor 2; *IDH*, Isocitrate Dehydrogenase gene.

**Table 5:** Summary of image techniques, methods, and features extracted in the reviewed studies to build predictive models on cancer prognosis.

Methods	Type of Data	Features	Used by
Image segmentation and hand-crafted features	WSIs	Quantitative image features	[27,28,37,48,54]
		ROIs from WSIs	[80,99,103,104,105,107]
	MRI images	Quantitative image features	[87]
	CT images	ROIs	[101,105]

Abbreviations: WSIs, Whole-Slide Images; MRI, Magnetic Resonance Imaging; CT, Computed Tomography; ROIs, Regions of Interest.

**Table 6:** Information related to the techniques used in the articles that applied a conventional statistical approach when building cancer prognosis predictive models.

First Author & Reference	Predictive Modelling	Validation Technique(s)	Performance Metrics	Model Output	Dimensionality Reduction	External Validation	Model Comparison
Zhu [68]	SuperPC regression	10-fold CV	HR and Log-rank tests *p*-value	HR. Dichotomization of patients into high/low-risk and low-risk	✔		
Cheng [69]	Lasso–Cox model	10-fold CV	Log-rank test *p*-value	Risk index of death	✔		
Dos Reis [70]	Multivariate CPH regression within a multivariable fractional polynomial model	No	AUC	Risk index of death at 10-years	✔	✔	✔
Sperduto [71]	Multivariate multiple CPH regression	No	None	Lung-molGPA score	✔		
Elwood [72]	Multivariate CPH regression	Bootstrapping for internal and external validation	C-index	Predicted OS (months) at 10 years		✔	✔
Matsuo [73]	Multivariate CPH regression	10-fold CV	MAE, C-index	Survival risk index, PFS, and OS			✔

Abbreviations: superPC, supervised Principal Components; CPH, Cox Proportional Hazards; CV, cross-validation; HR, Hazard Ratio; AUC, Area Under Curve; MAE, Median Absolute Error; OS, Overall Survival; PFS, Progression-Free Survival.

**Table 7:** Information related to the techniques used in the articles that applied machine learning techniques when building cancer prognosis predictive models.

First Author & Reference	Predictive Modelling	Validation Technique(s)	Performance Metrics	Model Output	Dimensionality Reduction	External Validation	Model Comparison
Matsuo [73]	DNN	10-CV	MAE, C-index	Predicted OS and PFS			✔
Mohebian [74]	BDT	Bagging, hold-out and 4-CV	*Sn*, *Sp*, *Acc*, precision, F-score, AUC, MCC, *+LR*, *-LR*, DOR, DP, κ	Patient dichotomization	✔		✔
Obrzut [75]	PNN, MLP, GEP, SVM, RBFNN, and K-means	10-CV	*Acc*, *Sn*, *Sp*, AUC	Predicted OS at 5 years			✔
Zhu [76]	MOK	Monte Carlo CV	C-index	Predicted overall prognostic score	✔	✔	✔
Chaudhary [61]	DL-based model	5-CV and 10-CV	C-index, log-rank *p*-value, and BS	Patient dichotomization	✔	✔	✔
Sun [77]	SimpleMKL	10-CV	AUC, *Acc*, precision, MCC, and C-index	Patient dichotomization	✔		✔
Zhang [78]	ANN, K-means, SVM, and XGBoost	10-CV	AUC	Predicted OS and patient dichotomization	✔	✔	✔
Zhao [79]	Gradient Boosting, RF, SVM, and ANN	10-CV	ROC curve, *Acc*, CS, stability	Patient dichotomization	✔		✔
Cheerla [80]	DNN	Hold-out	C-index	Predicted OS	✔		
Ferroni [81]	MKL based on SVM	3-CV	AUC, *Sn*, *Sp*, F- score, *LR*, HR, C-index, and *Acc*	Patient dichotomization			
Jing [82]	DNN	Bootstrapping	C-index	Predicted DFS and patient dichotomization		✔	✔
Sun [83]	DNN	10-CV	ROC curve, AUC, *Sn*, *Sp*, *Acc*, precision, MCC	Patient dichotomization	✔	✔	✔
Tapak [84]	NB, RF, AdaBoost, SVM, LS-SVM, AdaBag	Hold-out	*Sn*, *Sp*, PPV, NPV, *+LR*, *-LR*, *Acc*	Patient dichotomization			✔
Baek [85]	SVM, LR, L2RR, RF	Hold-out and 5-CV	*Acc*, AUC, C-index, IBS	Predicted DFS and OS at 5 years	✔		✔
Boeri [86]	SVM, ANN	3-CV	*Acc*, *Sn*, *Sp*, AUC	Risk of recurrence and risk of death	✔		
Choi [87]	RSF	Bagging	iAUC	Predicted OS and patient dichotomization	✔	✔	
Zhang [88]	MKL based on SVM	10-CV	AUC	Patient dichotomization	✔		✔
Arya [89]	Ensemble of CNNs and RF	10-CV	AUC, *Sn*, *Sp*, *Acc*, precision, MCC	Patient dichotomization	✔	✔	✔
Tong [90]	ANN	4-CV	C-index	HR	✔		
Owens [91]	DL-based model	Not detailed	Silhouette score, log-rank *p*-value	Patient dichotomization	✔	✔	
Malik [92]	DL-based model	10-CV	AUC, *Acc*, *Sn*, *Sp*, FPR, F1-Score, MCC, κ	Patient dichotomization	✔	✔	✔
Zhao [93]	ANN	10-CV	C-index	Patient dichotomization	✔		✔
Hassanzadez [94]	DL-based model	Hold-out and 5-CV	*Acc*	Patient dichotomization	✔		✔
Zhang [95]	DL-based model	Not detailed	C-index, IBS	Predicted OS	✔		✔
Chharia [96]	DL-based model	5-CV	Precision, *Acc*	Probability of survival and patient dichotomization	✔		✔

Abbreviations: BDT, Bagged Decision Tree; PNN, Probabilistic Neural Network; MLP, Multilayer Perceptron; GEP, Gene Expression Programming; RBFNN, Radial Basis Function Neural Network; MOK, Multi-Omic Kernel; DL, Deep Learning; DNN, Deep Neural Network; MKL, Multiple Kernel Learning; SVM, Support Vector Machine; NB, Naïve Bayes; LS-SVM, Least-Squares Support Vector Machine; LR, Logistic Regression; L2RR, L2 Regularised regression; RF, Random Forest; ANN, Artificial Neural Network; XGBoost, Extreme Gradient Boosting; RSF, Random Survival Forest; CV, cross-validation; Sn, Sensitivity; Sp, Specificity; Acc, accuracy; AUC, Area Under Curve; MCC, Matthews Correlation Coefficient; +LR, Positive Likelihood Ratio; -LR, Negative Likelihood Ratio; DOR, Diagnostic Odds Ratio; DP, Discriminant Power; κ, Cohen’s kappa coefficient; ROC, Receiving Operating Characteristics; CS, Calibration Slope; *LR*, Likelihood Ratio; FPR, False Positive Rate; HR, Hazard Ratio; MAE, Mean Absolute Error; PPV, Positive Predictive Value; NPV, Negative Predictive Value; BS, Brier Score; IBS, Integrated Brier Score; iAUC, integrated Area Under Curve; OS, Overall Survival; DFS, Disease-Free Survival; PFS, Progression-Free Survival.

**Table 8:** Information related to the techniques used in the articles that applied a mixed approach (conventional statistics together with machine learning) when building cancer prognosis predictive models.

First Author & Reference	Predictive Modelling	Validation Technique(s)	Performance Metrics	Model Output	Dimensionality Reduction	External Validation	Model Comparison
Yousefi [97]	DL-CPH	Monte Carlo CV	C-index	Risk index of death, correlated to OS			✔
Katzman [98]	DL-CPH	Bootstrapping, hold-out and 3-CV	C-index	HR		✔	✔
Mobadersany [99]	DL-CPH	Monte Carlo CV	C-index	HR			✔
Huang [100]	DL-CPH	5-CV	C-index, log-rank test *p*-value	HR and patient dichotomization	✔		✔
Wang [101]	DL-CPH	Not detailed	C-index, AUC, *Acc*, log-rank test *p*-value	Risk index of recurrence, correlated to RFS and Patient dichotomization. Recurrence probability in a specific time point		✔	✔
Shao [102]	Adaboost for diagnosis and CPH for prognosis	5-CV	C-index, BS	Risk index of death and patient dichotomization	✔		✔
Chen [103]	DL-CPH	15-CV	C-index	Patient dichotomization	✔		
Hao [104]	DL-CPH	Not detailed	C-index	Patient dichotomization	✔		✔
Ning [105]	DL-CPH	10-CV	C-index	Patient dichotomization	✔		
Chai [106]	DL-CPH	Not detailed	C-index	Patient dichotomization	✔	✔	✔
Vale-Silva [107]	DNN-based model	Hold-out	Ctd, IBS	Conditional survival probability for 1 to 30 years	✔		✔
Wang [108]	NMF-CPH	3-CV	C-index	Survival probability and patient dichotomization	✔	✔	✔
Poirion [109]	Ensemble of DL and SVM models	Hold-out and 5-CV	Log-rank *p*-value, C-index and Silhouette score	Patient’s risk of death	✔	✔	✔
Zhang [110]	DL-CPH	10-CV	AUC	Patient dichotomization	✔	✔	

Abbreviations: CPH, Cox Proportional Hazards; ML, Machine Learning; DL-CPH, Deep Learning Cox Proportional Hazard; DNN, Deep Neural Networks; NMF, non-negative matrix factorisation; CV, cross-validation; LOO-CV, Leave-One-Out Cross-Validation; AUC, Area Under Curve; *Acc*, accuracy; BS, Brier Score; Ctd, time-dependent concordance index; IBS, integrated Brier Score; OS, Overall Survival; HR, Hazard Ratio; RFS, Recurrence Free Survival.

**Table 9:** Summary of data sources used to build predictive models of cancer prognosis.

Type of Repository	Repositories & Programs/Studies	Used by
Public Repositories	ICGC Data Portal (e.g., Pan-Cancer Atlas Initiative, TCGA Program)	[61,69,76,77,80,83,85,87,88,89,90,91,92,93,94,95,97,99,100,102,103,104,105,106,107,108,109,110]
	EGA (e.g., METABRIC Study)	[79,82,83,89,96,98,109]
	GDC Data Portal (e.g., TARGET Program)	[61,76,78,95]
	GEO	[61,95,106,108,109,110]
	COSMIC	[85]
	ArrayExpress Archive of Functional Genomics Data	[61]
Institutional databases	N/A	[71,72,73,74,75,81,82,84,86,87,101]

Abbreviations: ICGC, International Cancer Genome Consortium; TCGA, The Cancer Genome Atlas; EGA, European Genome-Phenome Archive; METABRIC, Molecular Taxonomy of Breast Cancer International Consortium; GDC, Genomics Data Commons; GEO, Gene Expression Omnibus; COSMIC, Catalogue of Somatic Mutations in Cancer.
